# Results from a systematic review of interventions promoting mental health literacy in youth

**DOI:** 10.1093/eurpub/ckac129.248

**Published:** 2022-10-25

**Authors:** A Fretian, S Kirchhoff, O Okan, U Bauer

**Affiliations:** 1 Department of Sport and Health Science, Technical University Munich, Munich, Germany; 2 Faculty of Educational Science, Bielefeld University, Bielefeld, Germany

## Abstract

**Background:**

Mental illnesses are amongst the leading causes of ill-health and disability, with most onset of many mental health problems (ca. 75%) emerging before the age of 25 years. Thus, adolescence is an important time period for preventive measures, such as strengthening mental health literacy (MHL). This review aims to give an overview of those interventions which promote MHL on the long run.

**Methods:**

Five databases were searched for English or German articles published between January 1997 and May 2020, leading to a total of 4,375 original articles. Interventions were included only if they had measured MHL and/or stigma on three different time points, had a control group, and delivered an intervention program. Studies reporting means and standard deviations for the outcomes of interest were further included into a meta-analysis using a random effects model. The analysis was carried out with STATA 16.

**Results:**

25 studies were included into the review, and 13 of them were suitable for the meta-analysis. The great majority of studies (76%) were conducted within schools and the addressed topics were general mental health, depression, and schizophrenia. Interventions mostly used psychoeducation or a combination of educational elements and contact as delivery method. The combined use of educational and contact components led to worse results for mental health literacy, but not stigmatizing attitudes or social distance. Generally, interventions led to positive outcomes. The changes were sustained for mental health literacy d = 0.48, as well as for stigmatizing attitudes d = 0.30, and social distance d = 0.16, after an average follow-up of about 5 months.

**Conclusions:**

MHL-interventions targeting adolescents are mostly conducted within schools and generally have a brief follow-up period. They show a stable improvement in mental health literacy and are to a smaller degree able to destigmatize mental illness or improve social distance.

